# Irisin inhibits adipogenic differentiation of bone marrow mesenchymal stem cells through the SIRT1/RANBP2/FTO signaling axis and protects against osteoporosis

**DOI:** 10.1038/s41420-026-02976-5

**Published:** 2026-02-25

**Authors:** Junfei Chen, Jincheng Liu, Qingyang Fu, Mingyu Xu, Xu Zhai, Le Li, Wanlong Xu, Xinhui Wu, Kaidi Wang, Haipeng Si

**Affiliations:** 1 Department of Pediatric Surgery, Qilu Hospital of Shandong University, Cheeloo College of Medicine, Shandong University, Jinan, Shandong PR China; 2 Department of Orthopedics, Qilu Hospital of Shandong University, Jinan, Shandong PR China; 3 Department of Nephrology, Shandong Electric Power Central Hospital, Jinan, Shandong PR China; 4 Department of Orthopedics, Key Laboratory of Qingdao in Medicine and Engineering, Qilu Hospital of Shandong University (Qingdao), Qingdao, Shandong PR China; 5 Department of Medical Experimental Center, Qilu Hospital (Qingdao), Cheeloo College of Medicine, Shandong University, Qingdao, Shandong PR China

**Keywords:** Cell signalling, Sumoylation

## Abstract

Abnormal bone marrow obesity caused by the conversion of bone marrow mesenchymal stem cells (BMMSCs) from osteoblast to adipocyte differentiation is one of the significant contributors to age and menopause-related osteoporosis (OP) development. Irisin, one of the myokines, has been reported to be involved in skeletal metabolic diseases, providing new insights into the pathogenesis of OP. However, the specific mechanism of irisin in adipogenic differentiation of BMMSCs has not been thoroughly explored. Clinical data from this study confirmed the expression of irisin in OP and its clinical significance. In addition, irisin inhibited adipogenic differentiation of BMMSCs in vitro and reduced bone loss and abnormal bone marrow obesity in ovariectomized (OVX) mice. Mechanistically, SIRT1 was identified as a downstream target of irisin, and activated SIRT1 inhibited the expression of FTO through deacetylating RANBP2, which downregulated the stability and expression of PPARγ. The current study revealed a novel molecular mechanism by which irisin mediated BMMSCs adipogenesis through the SIRT1/RANBP2/FTO signaling axis.

## Introduction

Osteoporosis (OP) is a systemic metabolic bone disease characterized by low bone mass, decreased bone strength, damage of bone microstructure, and fracture [1–3]. OP can occur at any age, with postmenopausal women and older men being the most common [4, 5]. With the accelerated aging of the global population, the prevalence of osteoporosis is rapidly rising [6]. OP has become one of the major public health problems jeopardizing the health of the global elderly population, but lacking effective preventive and curative measures [7, 8]. Therefore, there is an urgent requirement to identify the underlying causes and better treatments for OP.

Abnormal differentiation of bone marrow mesenchymal stem cells (BMMSCs) is one of the important factors in the pathogenesis of postmenopausal OP [9, 10]. BMMSCs, mainly located in bone marrow, are mesoderm-derived stem cells with self-renewal and multidirectional differentiation ability, which can differentiate into osteoblasts or adipocytes and are intimately related to bone reconstruction [11, 12]. Hyperdifferentiation of BMMSCs toward adipocytes can result in a decrease in the number of osteoblasts and a relative increase in the number of osteoclasts, causing an imbalance in bone homeostasis [13, 14].

Irisin is a myogenic factor produced and secreted primarily by muscle and has a significant role in the regulation of energy metabolism, glycolipid homeostasis, and bone health status [15–17]. The precursor gene encoding irisin is the transmembrane fibronectin type III domain-containing protein 5 (FNDC5) [18]. The structural domains of FNDC5 consist of an N-terminal signal peptide, a type III fibronectin structural domain, a hydrophobic knot relaxation domain in the transmembrane region, and an intracellular C-terminal structural domain [17, 19]. The FNDC5 protein is hydrolyzed by proteases to produce irisin containing 112 amino acids [17, 20].

In recent years, it has been shown that irisin has a protective effect on the skeleton and serves as a predictor of fragility fractures in osteoporosis as well as a biomarker for determining prognosis [21, 22]. Some studies have found that irisin could activate the Wnt/B-catenin signaling pathway by increasing autophagy-related proteins, thereby promoting the differentiation of BMMSCs to osteoblasts [23]. In addition, reports have demonstrated that irisin may decelerate the progression or worsening of fatty liver-related diseases through its antilipidemic effects [24, 25]. Park et al. demonstrated that irisin suppressed palmitic acid-induced hepatic lipogenesis and lipid overaccumulation by modulating the protein arginine methyltransferase-3 and altering the expression of adipogenic genes, providing a theoretical basis for the prevention of steatosis by irisin [26]. However, the specific mechanism of irisin on the differentiation of BMMSCs to adipocytes has not been elucidated.

As a member of the NAD+-dependent protein deacetylase family, Sirtuin1 (SIRT1) catalyzes the deacetylation of histones as well as other substrates including p53, nuclear factor κB (NF-κB), FoxOs (forkhead box O family), and Ku70 [27–31]. SIRT1 participates in a wide variety of biological processes, including DNA repair, apoptosis, senescence, inflammation, metabolism, and autophagy [32, 33]. Studies have shown that activated SIRT1 promoted bone repair by enhancing the coupling of H-type angiogenesis and bone formation through the PI3K/AKT/FOXO1 signaling pathway [34]. Huang et al. found that melatonin regulated the osteogenic and adipogenic differentiation of BMMSCs by activating the SIRT1 signaling pathway [35]. However, it has not been established whether the SIRT1-related signaling pathway is involved in determining BMMSCs lineage fate by irisin.

In this study, we performed a comprehensive analysis using cellular and animal models, as well as a clinic-based sample study to further substantiate the potential clinical use of irisin in the treatment of postmenopausal OP. Our study revealed that irisin could affect the adipogenic differentiation of BMMSCs by modulating the SIRT1/RANBP2/FTO signaling axis, while in vivo experiments indicated that irisin ameliorated the progression of postmenopausal OP by decreasing the excessive adipogenicity of the bone marrow cavity. Overall, the present study highlighted the potential mechanism of irisin-mediated SIRT1 regulation of adipogenic differentiation in BMMSCs and may provide new insights into diagnostic and therapeutic strategies for OP.

## Results

### Irisin level was negatively correlated with adipogenic differentiation of BMMSCs and was decreased in the serum of osteoporotic mice or osteoporotic patients

**Fig. 1:**
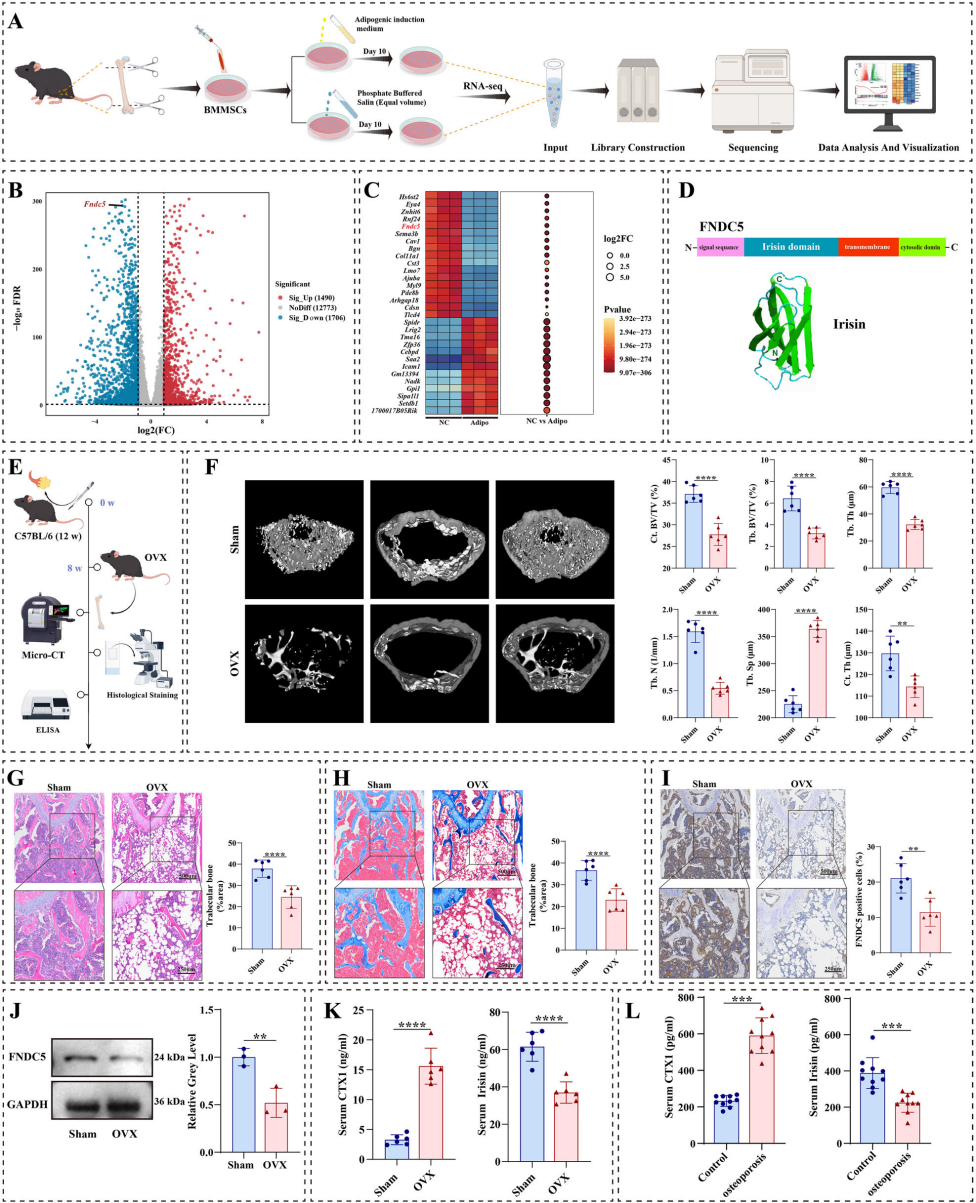
Irisin level was negatively correlated with adipogenic differentiation of BMMSCs and was decreased in the serum of osteoporotic patients or osteoporotic mice. **A** Schematic workflow of the RNA-seq. **B** Volcanic maps of RNA-Seq during adipogenic differentiation in BMMSCs. **C** Heatmap of mRNA expression during adipogenic differentiation, with high and low expression levels shown in red and blue, respectively. **D** Structure of FNDC5 and irisin. **E** Schematic diagram of the OVX modeling workflow. **F** Representative micro-CT images of trabecular bone from the femoral metaphysis in sham and OVX mice. Cortical bone volume/total volume (Ct. BV/TV), trabecular bone volume/total volume (Tb. BV/TV), trabecular thickness (Tb. Th), cortical thickness (Ct. Th), and number of trabeculae (Tb. N), and trabecular separation (Tb. Sp) analysis of the femurs in sham and OVX mice. **G** Representative images of H&E staining in sham and OVX mice. **H** Representative images of Masson staining in sham and OVX mice. **I** Representative images of immunohistochemistry staining for FNDC5 in sham and OVX mice. **J** FNDC5 expression in bone tissue was detected by western blotting in the Sham and OVX mice. **K** The serum concentration of CTX-1 and irisin was detected by ELISA in the sham and OVX mice. **L** The serum concentration of CTX-1 and irisin was detected by ELISA in the control group and osteoporosis group. The values are mean ± SD of at least three independent experiments; n.s.*p* > 0.05, ^*^p < 0.05, ^**^p < 0.01, ^***^p < 0.001, ^****^p < 0.0001.

We performed RNA-seq to visualize differential genes during adipogenic differentiation of BMMSCs (Fig. 1A). Next, we analyzed the differentially expressed genes in RNA-seq (|FC| > 2 and *p* < 0.05), of which the number of differentially expressed genes in RNA-seq was 3196 (Fig. 1B). The Clustered heatmap showed the top 30 differential genes, and the expression of FNDC5, the precursor linker protein of irisin, was significantly reduced during adipogenic differentiation of BMMSCs (Fig. 1C, D). Subsequently, we constructed OVX mice for in vivo experiments (Fig. 1E). Micro-computed tomography (Micro CT) analysis exhibited a decrease in cortical bone volume/total volume (Ct. BV/TV), trabecular bone volume/total volume (Tb. BV/TV), trabecular thickness (Tb. Th), cortical thickness (Ct. Th), and the number of trabeculae (Tb. N), and a significant increase in trabecular separation (Tb. Sp) compared with the sham group (Fig. 1F). H&E and Masson staining indicated bone loss in OVX mice (Fig. 1G, H). Immunohistochemical staining showed a significant reduction in FNDC5 protein positivity in bone tissue of OVX mice (Fig. 1I). Western blotting results demonstrated that the FNDC5 protein level was significantly lower in bone tissue of OVX mice (Fig. 1J). In addition, OVX mice displayed reduced serum levels of irisin and elevated levels of CTX1 (Fig. 1K). According to the bone mineral density (BMD) determined by dual-energy X-ray absorptiometry (DXA), we obtained 20 bone tissue samples and serum samples from osteoporotic and non-osteoporotic patients, respectively, and then aggregated the baseline information of the patients (Table S3). Consistent with the results obtained with OVX mice serum samples, serum irisin level was significantly lower in the OP patients, and CTX1 expression showed the inverse trend (Fig. 1L). Thus, our clinical data and in vivo findings suggested that osteoporosis may be associated with a significant decrease in irisin levels.

### Irisin inhibited adipogenic differentiation of BMMSCs in vitro

**Fig. 2:**
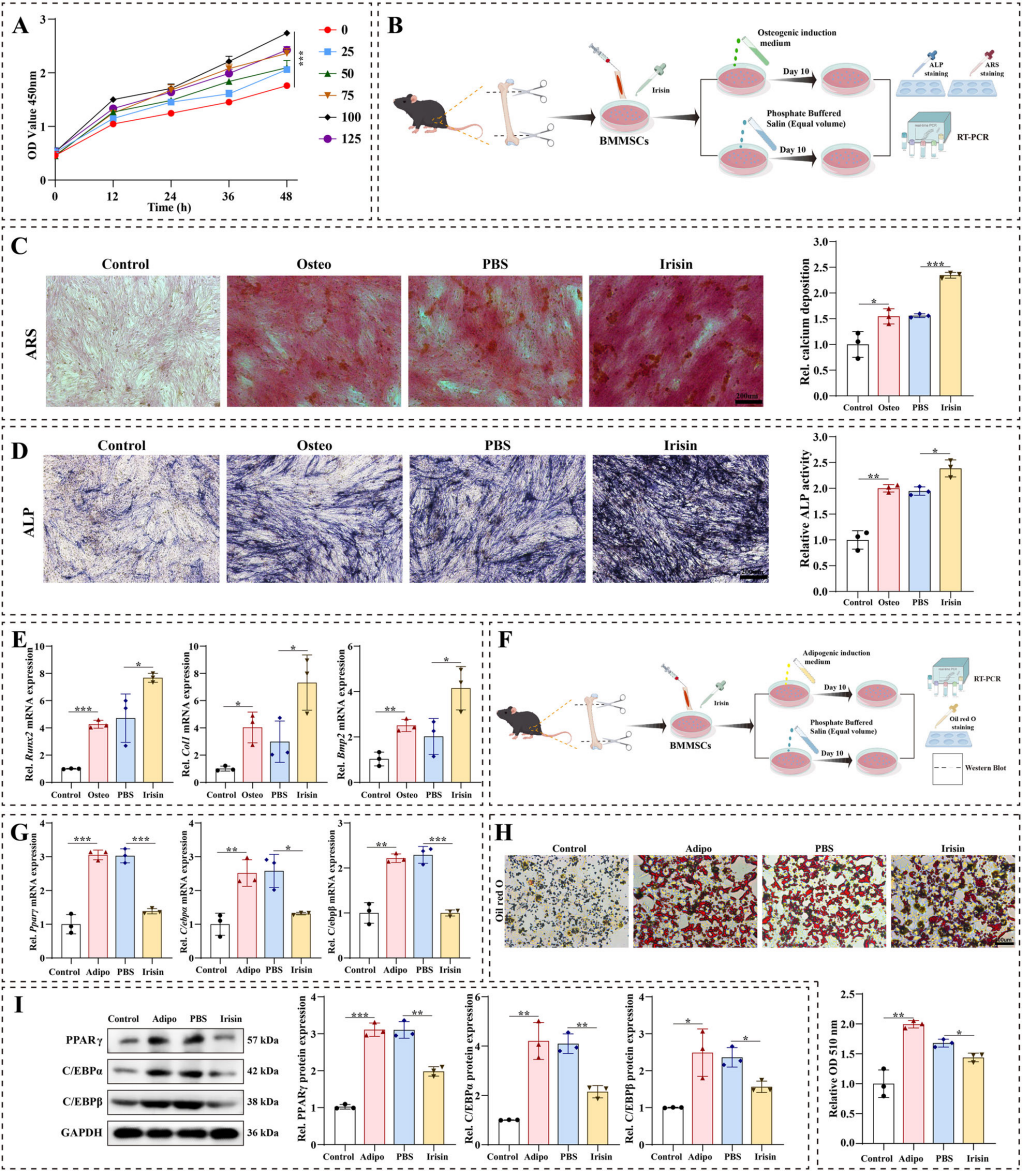
Irisin regulated differentiation of BMMSCs in vitro. **A** The effects of 10 μM, 25 μM, 50 μM, 75 μM, 100 μM, and 125 μM concentrations of irisin on the proliferation of BMMSCs were detected on days 12 h, 24 h, 36 h, and 48 h using the CCK-8 assay. **B** Flow chart of experiments to detect the osteogenic differentiation of BMMSCs. **C** Representative images of ARS staining during osteogenic (Osteo) differentiation in BMMSCs, and quantitative analysis of ARS staining. **D** Representative images of ALP staining during osteogenic (Osteo) differentiation in BMMSCs, and quantitative analysis of ALP staining. **E** After the addition of irisin, qRT-PCR was performed to analyze the mRNA levels of osteogenic (Osteo) related genes (*Runx2*, *Col1*, and *BMP2*) in BMMSCs during the osteogenic differentiation. **F** Flow chart of experiments to detect the adipogenic differentiation of BMMSCs. **G** After the addition of irisin, qRT-PCR was performed to analyse the mRNA levels of adipogenic (Adipo) related genes (*Pparγ*, *C/ebpα*, and *C/ebpβ*) in BMMSCs during the adipogenic differentiation. **H** Representative images of Oil red O staining after adding irisin, and quantitative analysis of Oil red O staining. **I** After the addition of irisin, the levels of adipogenic (Adipo) related protein (PPARγ, C/EBPα, and C/EBPβ) in BMMSCs during the adipogenic differentiation were measured by western blotting. The values are mean ± SD of at least three independent experiments; n.s.*p* > 0.05, ^*^p < 0.05, ^**^p < 0.01, ^***^p < 0.001, ^****^p < 0.0001.

To further validate the biological function of irisin on the differentiation of BMMSCs, we used exogenous irisin to treat BMMSCs in the process of adipogenesis. First, the CCK-8 assay was used to evaluate the effect of different concentrations of irisin on the BMMSCs proliferation. The results showed that the ability of irisin to promote the proliferation of BMMSCs was enhanced with the increase of irisin concentration (0, 25, 50, 75, and 100 ng/mL), but the proliferative ability of BMMSCs was decreased when the irisin concentration was increased to 125 ng/mL (Fig. 2A). Therefore, to better simulate the effect of irisin-induced differentiation of BMMSCs, we chose a concentration of 100 ng/ml of irisin in the subsequent experiments. Previous studies showed that irisin played an important role in the osteogenic differentiation of BMMSCs [36, 37]. Firstly, we conducted a series of functional experiments to verify the biological function of irisin in the osteogenic differentiation of BMMSCs (Fig. 2B). Irisin (100 ng/ml) was added to BMMSCs during osteogenic differentiation. ARS staining indicated that irisin promoted the formation of mineralized nodules after osteogenic induction in BMMSCs (Fig. 2C). Similarly, the results of ALP staining displayed that irisin improved the osteogenic differentiation ability of BMMSCs compared with the PBS group (Fig. 2D). qRT-PCR results also demonstrated that the expression of genes related to osteogenic differentiation, including Runt-related transcription factor 2 (*Runx2*), Collagen I (*Col1*), and bone morphogenetic protein 2 (*Bmp2*), were increased after irisin addition (Fig. 2E). Consistent with previous studies, our study found that irisin indeed promoted the osteogenic differentiation of BMMSCs. Then, we performed a series of functional experiments to verify the biological function of irisin in the adipogenic differentiation of BMMSCs (Fig. 2F). Irisin (100 ng/ml) was added to BMMSCs during adipogenic differentiation. mRNA levels of genes related to adipogenic differentiation, including peroxisome proliferators-activated receptors *γ* (*Pparγ*), CCAAT/enhancer binding protein *α* (*C/ebpα*), and CCAAT/enhancer binding protein *β* (*C/ebpβ*), were decreased after irisin addition (Fig. 1G). In addition, Oil red O staining showed that irisin addition decreased the number of lipid droplets in the adipogenic differentiation of BMMSCs (Fig. 2H). Consistently, western blotting confirmed that irisin treatment significantly reduced PPARγ, C/EBPα, and C/EBPβ protein expression during adipogenic differentiation of BMMSCs (Fig. 2I, J). These results suggested that irisin had significant inhibitory effects on adipogenic differentiation and enhanced osteogenic potential in BMMSCs.

### Irisin rescued bone loss and inhibited excessive adipogenesis in bone marrow tissue in OVX mice

**Fig. 3:**
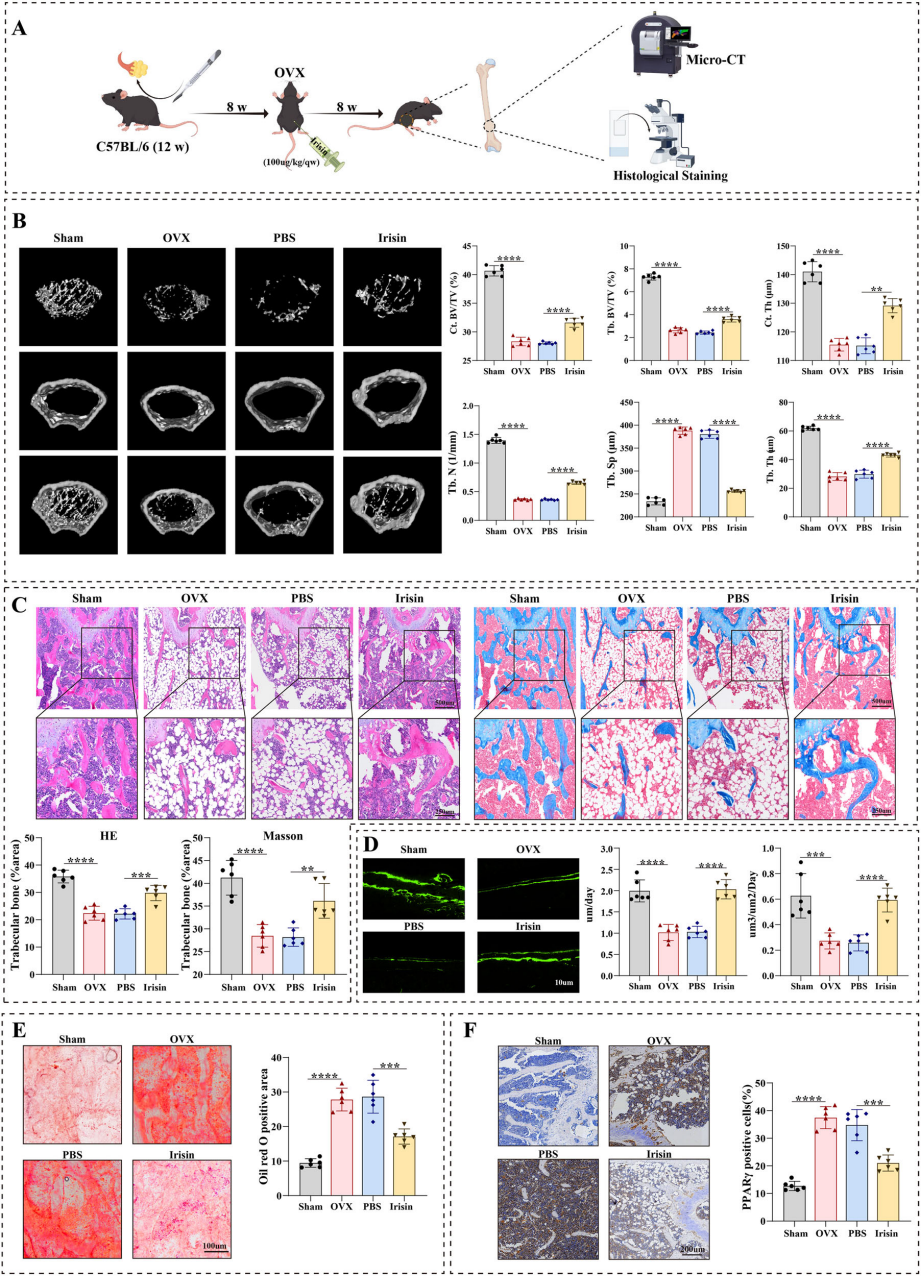
Irisin rescued bone loss and inhibited excessive adipogenesis in bone marrow tissue in OVX mice. **A** Flowchart of the experimental procedure for intraperitoneal injection of irisin in OVX mice. **B** Representative micro-CT images of trabecular bone from the distal femur. Micro-CT measurements of Ct.BV/TV, Tb.BV/TV, Tb.Th, Ct.Th, Tb. N, and Tb.Sp in the femur after treatment with PBS or irisin. (**C**) Representative images of H&E staining and Masson staining from the distal femur in PBS and irisin groups. H&E staining and Masson staining of quantitative analysis in PBS and irisin groups. (**D**) Calcein double label staining image and quantitative analysis of MAR and BFR/BS in PBS and irisin groups. (**E**) Representative images of Oil red O staining in the distal femur, and quantitative analysis of Oil red O staining. (**F**) Representative images of immunohistochemistry staining for PPARγ in PBS and irisin groups, and quantitative analysis of immunohistochemistry staining. The values are mean ± SD of at least three independent experiments; n.s.*p* > 0.05, ^*^p < 0.05, ^**^p < 0.01, ^***^p < 0.001, ^****^p < 0.0001.

In vivo, tests were used to further determine the protective effect of irisin against bone loss in OVX mice. Irisin was intraperitoneally administered to OVX mice at a dose of 100 μg/kg once a week (Fig. 3A). Micro-CT analysis showed a significantly higher bone mass (higher Ct. BV/TV and Tb. BV/TV) and upregulated trabecular bone structure (higher Tb. Th and Tb. N, and lower Tb. Sp) in the irisin group compared with PBS group (Fig. 3B). H&E staining and Masson staining of the femur consistently also confirmed that irisin significantly increased capacity for bone formation in vivo (Fig. 3C). Additionally, calcein double-label staining results demonstrated that irisin facilitated the formation of new bone in cortical bone (Fig. 3D). Consistent with the results of the in vitro experiments, Oil red O staining of the bone marrow cavity of the femur showed that irisin significantly reduced the number of lipid droplets and the area of bone marrow fat, suggesting that irisin played a positive role in inhibiting the ability of adipogenic differentiation to promote bone formation in OVX mice (Fig. 3E). Immunohistochemical staining showed a significant reduction in PPARγ protein positivity in the bone tissue of irisin-treated OVX mice (Fig. 3F). We found that irisin rescued bone loss in OVX mice by inhibiting bone marrow fat accumulation.

### Irisin regulated the adipogenic differentiation of BMMSCs by activating *Sirt1*

**Fig. 4:**
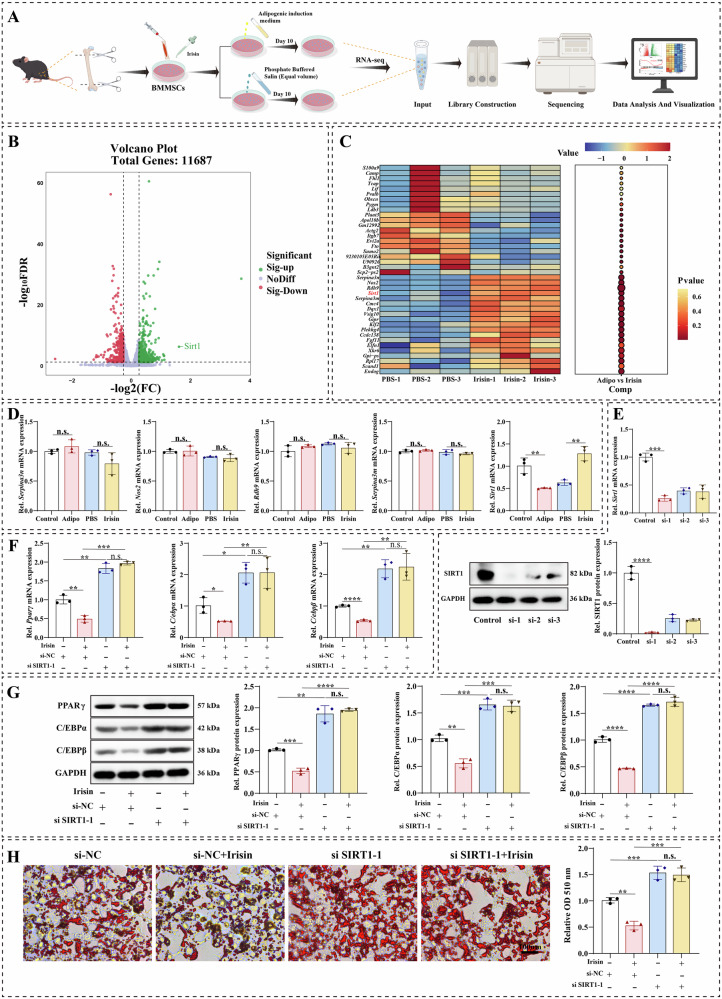
Irisin regulated the adipogenic differentiation of BMMSCs by activating *Sirt1.* **A** Schematic workflow of the RNA-seq. **B** Volcanic maps of RNA-Seq in the PBS group and irisin group. **C** Heatmap of mRNA expression in the PBS group and irisin group, with high and low expression levels shown in red and blue respectively. **D** The mRNA levels of *Serpina3n*, *Nos2*, *Rdh9*, *Serpina3m*, and *Sirt1* were detected by qRT-PCR. **E** qRT-PCR and western blotting confirmation of *Sirt1* knockdown BMMSCs. **F** mRNA expressions of *Pparγ*, *C/ebpα*, and *C/ebpβ* in *Sirt1* knockdown cells with or without irisin treatment. **G** Protein expressions of PPPARγ, C/EBPα, and C/EBPβ in *Sirt1* knockdown cells with or without irisin treatment. **H** Representative images of Oil red O staining in *Sirt1* knockdown cells with or without irisin treatment, and quantitative analysis of Oil red O staining. The values are mean ± SD of at least three independent experiments; n.s.*p* > 0.05, ^*^p < 0.05, ^**^p < 0.01, ^***^p < 0.001, ^****^p < 0.0001.

To elucidate the downstream molecular mechanisms of irisin-mediated adipogenic differentiation of BMMSCs, we performed RNA-seq to detect differential genes between irisin-treated and untreated groups during adipogenic differentiation of BMMSCs (Fig. 4A). Based on RNA-seq, the volcano plot showed that irisin resulted in significant changes in genes during adipogenic differentiation of BMMSCs, identifying 530 genes with increased expression and 387 genes with decreased expression (Fig. 4B). The clustered heatmap showed the top 40 differential genes with a large number of differences (Fig. 4C). *Serpina3n*, *Nos2*, *Rdh9*, *Serpina3m*, and *Sirt1* were the top five significantly upregulated genes during adipogenic differentiation of BMMSCs. qRT-PCR analysis was used to validate the expression of these five genes, showing that *Sirt1* was the only gene specifically upregulated in the irisin-treated group compared to the PBS group (Fig. 4D). We transfected BMMSCs with *Sirt1* siRNA to attenuate its intracellular expression. Due to the highest knockdown efficiency, si*Sirt1*-1 was used for subsequent functional experiments (Fig. 4E). Next, the *Sirt1* plasmid was transfected with BMMSCs. qRT-PCR and Western blotting results showed that the expression of SIRT1 was markedly elevated after transfection (Fig. S1A). The transcript levels of adipogenesis-related genes were upregulated by *Sirt1* knockdown, and the *Sirt1* overexpression reversed the trend (Fig. S1B). Western blotting results indicated that *Sirt1* downregulation increased the expression of adipogenic-related proteins, including PPARγ, C/EBP α, and C/EBP β, and *Sirt1* overexpression diminished the levels of adipogenesis-related proteins (Fig. S1C). Oil red O staining showed that Sirt1 downregulation increased the adipogenic capacity of BMMSCs, whereas *Sirt1* increase minimized the number of lipid droplets during adipogenesis in BMMSCs (Fig. S1D). To further characterize whether irisin could mediate the adipogenic differentiation of BMMSCs by regulating *Sirt1*, we performed a series of recovery experiments. qRT-PCR and western blotting results revealed that irisin could decrease the indicators related to adipogenic differentiation, but the *Sirt1* knockdown decreased the inhibitory effect of irisin on adipogenic differentiation, and irisin had no effect on the upregulation in adipogenic capacity caused by *Sirt1* knockdown (Fig. 4F, G). Oil red O staining showed that the inhibitory effect of irisin on lipid droplet formation during adipogenic differentiation of BMMSCs was reversed by *Sirt1* knockdown (Fig. 4H).

It has been reported that irisin mediates skeletal biological functions via αV integrin receptors. Therefore, we further investigated whether irisin regulates SIRT1 expression through αV integrin receptors [38]. qRT-PCR results demonstrated that irisin significantly upregulated *Sirt1* expression, whereas the addition of cilengitide (an αV integrin receptor inhibitor) did not abrogate this inductive effect of irisin on SIRT1 (Fig. S2A). Collectively, these findings suggest that irisin may regulate SIRT1 expression through other mediators rather than αV integrin receptors.

Furthermore, we further investigated whether irisin regulates the progression of osteoporosis (OP) through Sirt1 in vivo. Micro-CT analysis revealed that irisin attenuated OVX-induced bone loss, whereas selisistat (a SIRT1 inhibitor) reduced this bone-protective effect of irisin (Fig. S3A). Oil Red O staining results demonstrated that compared with the irisin group, the addition of selisistat significantly increased the number of lipid droplets and the area of bone marrow adipocytes (Fig. S3B). These results further support that the regulatory effect of Irisin on BMMSCs is dependent on the involvement of SIRT1.

### Irisin regulated BMMSC adipogenic differentiation by SIRT1/FTO signaling axis

**Fig. 5:**
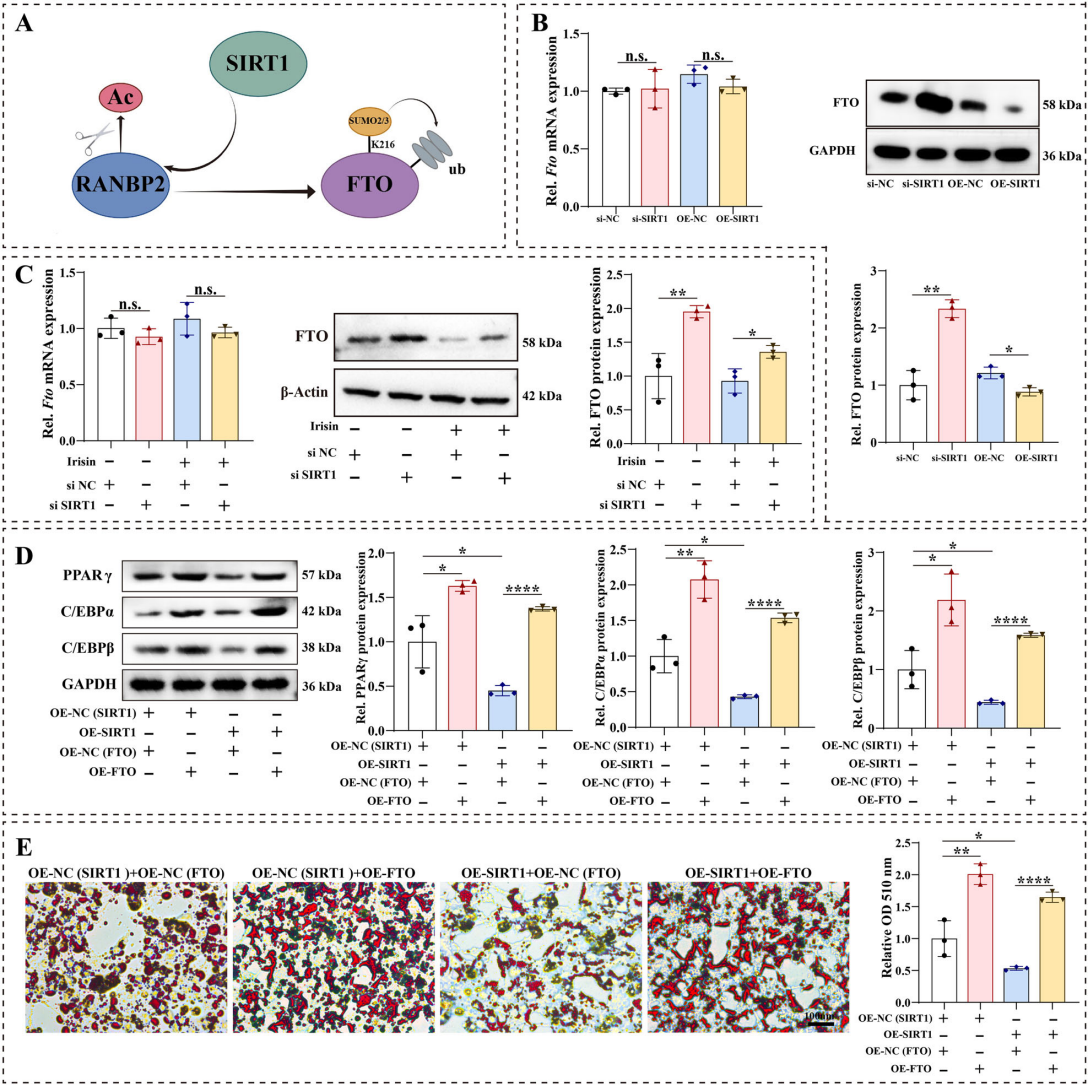
Irisin regulated BMMSC adipogenic differentiation by SIRT1/FTO signaling axis. **A** Flowchart of SIRT1 destabilized FTO via RANBP2-mediated SUMOylation. **B** *Fto* mRNA expression in *Sirt1* knockdown or *Sirt1*-overexpressed cells was assayed by qRT-PCR. *FTO* protein expression in *Sirt1* knockdown or *Sirt1*-overexpressed cells was assayed by western blotting. **C** *Fto* mRNA expression in *Sirt1* knockdown cells with or without irisin treatment was assayed by qRT-PCR. FTO protein expression in *Sirt1* knockdown cells with or without irisin treatment was assayed by western blotting. **D** Immunoblotting of adipogenic related proteins (PPARγ, C/EBPα, and C/EBPβ) in *Sirt1*-overexpressed cells with or without *Fto* overexpression. **E** Representative images of Oil red O staining in *Sirt1*-overexpressed cells with or without *Fto* overexpression, and quantitative analysis of Oil red O staining. The values are mean ± SD of at least three independent experiments; n.s.*p* > 0.05, ^*^p < 0.05, ^**^p < 0.01, ^***^p < 0.001, ^****^p < 0.0001.

Some studies have proposed that SIRT1 destabilized FTO via RANBP2-mediated SUMOylation [39] (Fig. 5A). The FTO protein, a member of the AlkB family of non-heme Fe(II)/dioxygenase 1 and the first reported gene linked to non-syndromic human obesity, plays a key role in adipogenesis and energy homeostasis by regulating fatty acid mobilization in adipocytes [40, 41]. Firstly, we verified the effect of SIRT1 on the expression of FTO in BMMSCs. Western blotting analysis demonstrated that *Sirt1* knockdown significantly increased the FTO level, whereas *Sirt1* overexpression suppressed its expression (Fig. 5B). However, the qRT-PCR results showed that SIRT1 did not alter the changes in FTO at the transcriptional level in BMMSCs (Fig. 5B). To further unravel the molecular mechanisms of irisin-mediated SIRT1 and FTO in the adipogenic differentiation of BMMSCs, we conducted a series of functional restoration experiments. Consistent with the previous trend, irisin-mediated SIRT1 did not alter the changes in FTO at the transcriptional level in BMMSCs (Fig. 5C). Western blotting analysis revealed that *Sirt1* knockdown reversed the reduction of FTO protein expression caused by irisin (Fig. 5C). Based on the above results, we further explored whether SIRT1 could mediate FTO to regulate the adipogenic differentiation of BMMSCs. Firstly, we detected the transfection efficiency of the overexpressed FTO plasmid in BMMSCs cells. qRT-PCR and western blotting results showed that FTO expression was significantly elevated after transfection (Fig. S4A). Subsequently, we transfected the FTO overexpression plasmid into BMMSCs with SIRT1 overexpression. qRT-PCR and western blotting demonstrated that SIRT1 upregulation attenuated the expression of adipogenic markers (PPARγ, C/EBPα, and C/EBPβ), while *Fto* overexpression significantly reversed this trend (Figs. S4B and 5D). Oil red O staining displayed that *Sirt1* overexpression significantly decreased the number of lipid droplets in adipogenic differentiation of BMMSCs, whereas *Fto* overexpression increased the ability to form lipid droplets (Fig. 5E).

### Irisin/SIRT1 inhibited FTO expression via RANBP2-mediated SUMOylation

**Fig. 6:**
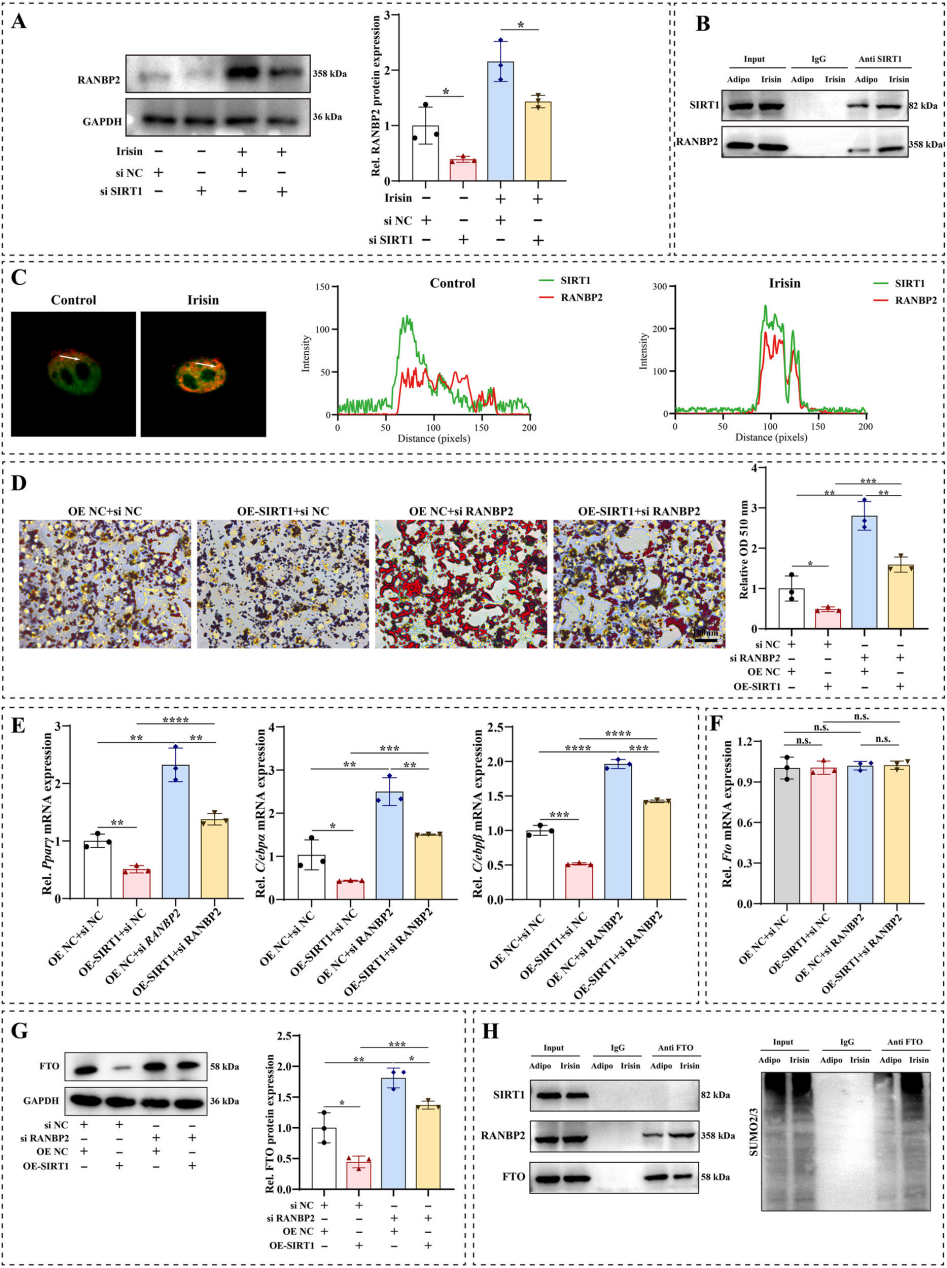
Irisin-mediated SIRT1 inhibited FTO expression via RANBP2-mediated SUMOylation. **A** RANBP2 protein expression in *Sirt1* knockdown cells with or without irisin treatment was assayed by western blotting. **B** Co-immunoprecipitation of SIRT1 with RANBP2 in BMMSCs with or without irisin treatment. **C** Colocalization of SIRT1 and RANBP2 after treatment with or without irisin in BMMSCs was exhibited in confocal immunofluorescent images. **D** Representative images of Oil red O staining in *Sirt1*-overexpressed cells with or without *Ranbp2* knockdown, and quantitative analysis of Oil red O staining. **E** mRNA expressions of *Pparγ*, *C/ebpα*, and *C/ebpβ* in *Sirt1*-overexpressed cells with or without *Ranbp2* knockdown. **F** *Fto* mRNA expression in *Sirt1*-overexpressed cells with or without *Ranbp2* knockdown was assayed by qRT-PCR. **G** FTO protein expression in *Sirt1-overexpressed* cells with or without *Ranbp2* knockdown was assayed by western blotting. **H** Co-immunoprecipitation was used to detect the interactions of FTO with SIRT1 and RANBP2 proteins in irisin-treated or non-treated BMMSCs. Co-immunoprecipitation was used to detect the interactions of FTO with SUMO2/3 in irisin-treated or non-treated BMMSCs. The values are mean ± SD of at least three independent experiments; n.s.*p* > 0.05, ^*^p < 0.05, ^**^p < 0.01, ^***^p < 0.001, ^****^p < 0.0001.

SIRT1 could enhance RAN binding protein 2 (RANBP2) stability through its deacetylating property [39]. Thus, we further explored whether irisin-mediated SIRT1 could regulate RANBP2 expression during adipogenic differentiation of BMMSCs. Irisin caused a significant increase in RANBP2 protein level, whereas *Sirt1* knockdown significantly reduced the promotion of RANBP2 by irisin (Fig. 6A). Irisin significantly increased *Ranbp2* mRNA expression, whereas downregulation of Sirt1 had no impact on *Ranbp2* mRNA levels (Fig. S5A). Irisin caused a significant increase in RANBP2 protein level, whereas *Sirt1* knockdown significantly reduced the promotion of RANBP2 by irisin (Fig. 6A). Moreover, the results of co-immunoprecipitation showed that SIRT1 specifically bound RANBP2 in irisin-mediated adipogenic differentiation in BMMSCs (Fig. 6B). Additionally, the result of confocal imaging demonstrated a great upregulation in the binding capacity of SIRT1 to RANBP2 after treatment with irisin in BMMSCs (Fig. 6C). Subsequently, we performed a series of functional recovery assays to confirm the interaction between SIRT1 and RANBP2 in the adipogenic differentiation of BMMSCs. We transfected *Sirt1* overexpression cells with si*Ranbp2*. Oil red O staining indicated that *Sirt1* overexpression significantly decreased the number of lipid droplets in adipogenic differentiation of BMMSCs, whereas *Ranbp2* knockdown increased the ability to form lipid droplets (Fig. 6D). *Sirt1* overexpression significantly decreased the expression of adipogenic markers (*Pparγ*, *C/ebpα*, and *C/ebpβ*), while *Ranbp2* knockdown significantly reversed this trend (Fig. 6E). The deacetylase SIRT1 is a crucial regulator of FTO downregulation via RANBP2-mediated SUMOylation. We further explored whether irisin-mediated SIRT1 could affect FTO expression via RANBP2 in the adipogenic differentiation of BMMSCs. Western blotting assays suggested that *Ranbp2* knockdown reversed the inhibition of FTO by *Sirt1* overexpression. However, the qRT-PCR results showed that *Ranbp2* knockdown did not alter the changes in FTO at the transcriptional level in *Sirt1* overexpression BMMSCs (Fig. 6F, G). Using co-immunoprecipitation to validate the interactions between SIRT1, RANBP2, and FTO, the results demonstrated that irisin enhanced the specific binding of RANBP2 to FTO, whereas SIRT1 did not bind specifically to FTO during irisin-mediated adipogenic differentiation, suggesting that FTO was not a direct transcription or deacetylation target of SIRT1 (Fig. 6H). In addition, the results of co-immunoprecipitation demonstrated that the SUMOylation of FTO was enhanced by irisin. The above results confirmed that irisin-mediated FTO influenced adipogenic differentiation of BMMSCs via SIRT1/RANBP2-mediated SUMOylation.

## Discussion

Abnormal amplification of bone marrow adipose tissue (MAT) plays a crucial role in the development of postmenopausal OP [35]. BMMSCs are precursor cells for osteoblasts and adipocytes in bone marrow [12]. In postmenopausal OP, the shift of BMMSCs from osteogenic to adipogenic differentiation contributes to impaired bone formation and increased accumulation of bone MAT [35, 42]. Estrogen supplementation resulted in decreased bone marrow obesity and increased bone mass in postmenopausal women and OVX mice, suggesting that MAT is associated with estrogen withdrawal-induced bone loss [43]. Therefore, aiming at MAT resulting from overdifferentiation of BMMSCs toward adipogenicity is a promising approach to ameliorate the development of postmenopausal OP. Currently, our study demonstrates for the first time the important mechanism whereby irisin-mediated SIRT1 destabilizes FTO, thereby attenuating adipogenic differentiation of BMMSCs to inhibit postmenopausal OP development. Our study has 3 noteworthy contributions.

Firstly, based on clinical samples and the OVX mice model, we found that irisin expression was downregulated in serum in postmenopausal OP. Next, we found that irisin inhibited adipogenic differentiation of BMMSCs and rescued bone loss in OVX mice. Finally, Irisin inhibited the adipogenic differentiation of BMMSCs by modulating the SIRT1/RANBP2/FTO signaling axis, thereby ameliorating OP. These findings suggested that irisin and its mediated molecular pathways involved in the adipogenic differentiation of BMMSCs could be a potential therapeutic target for OP.

Irisin, which is secreted by the muscles during exercise, plays a vital role in bone health [44]. It has been shown that irisin promoted osteoblast proliferation, differentiation, and mineralization mainly through the AMPK signaling pathway [45]. Consistent with our findings, as shown in Fig. 2C–G, we found that irisin enhanced the biological function of BMMSCs osteogenic differentiation. In addition, irisin-mediated JNK, Wnt/β-catenin, and RANKL/RANK/OPG signaling pathways regulated osteoclast differentiation and maturation [44, 46]. Irisin has been systematically studied in osteoblast-mediated osteogenic differentiation and osteoclast-mediated osteoclastic differentiation. However, as one of the key factors affecting OP, the specific mechanism of irisin in the adipogenic differentiation of BMMSCs remains unclear. Our findings that irisin gain-of-function prevented estrogen deficiency-induced postmenopausal OP and over-differentiation of BMMSCs toward adipogenesis established irisin as indispensable in defining the differentiation fate of BMMSCs, thereby ensuring bone health. Thus, our study supported the role of irisin-mediated modulation of adipogenic differentiation of BMMSCs in postmenopausal OP.

SIRT1 is a member of the NAD+-dependent deacetylase family, which is an important protective agent against oxidative stress and can be involved in the regulation of material energy metabolism, which is manifested in the regulation of the efficiency of glucose and lipid metabolism, the activity of mitochondrial biofunctions, and hormone metabolism levels [47–49]. In recent years, more and more studies have reported that SIRT1 participates in the regulation of skeletal metabolic disorders [50, 51]. In addition, SIRT1 promoted fatty acid catabolism and inhibited adipogenesis by inhibiting the transcription of PPARγ, fatty acid binding protein, and nuclear receptor corepressor [52, 53]. Our results revealed that irisin could inhibit the adipogenic differentiation of BMMSCs by activating SIRT1, and the inhibitory effect of irisin on adipogenesis of BMMSCs was reversed by *Sirt1* knockdown, which further suggested that SIRT1 was a downstream target gene of irisin in BMMSCs. Sun et al. reported that *Sirt1* overexpression reduced the acetylation level of FOXO3a in BMMSCs, inhibited osteoblast oxidative stress and senescence, promoted bone formation, and suppressed bone resorption in Bmi-1-deficient mice [54]. Our study revealed that irisin-mediated SIRT1 increased RANBP2 expression by exerting a biological function of deacetylation, thereby inhibiting adipogenic differentiation of BMMSCs. Our findings provided a novel model of irisin-mediated regulation in BMMSCs.

FTO proteins belong to the AlkB family of non-heme Fe (II)/dioxygenase 1, which is the first gene reported to cause non-syndromic human obesity [40, 55, 56]. Wang et al. identified that FTO modulated fatty acid mobilization in adipocytes by regulating *Angptl4* transcription to regulate body weight, suggesting that FTO played a key role in adipogenesis and energy homeostasis [41]. Our study found that Irisin could inhibit adipogenesis in BMMSCs by attenuating the expression of FTO protein. Shen et al. reported that FTO bound and demethylated PPARγ mRNA, leading to an increase in PPARγ mRNA expression, which promoted adipogenic differentiation of BMMSCs [57]. Our results demonstrated that irisin inhibited FTO protein level thereby significantly downregulating PPARγ expression and reducing adipogenic differentiation of BMMSCs, whereas the inhibitory effect of irisin on adipogenic differentiation of BMMSCs was reversed by FTO overexpression. It was reported that RANBP2, a key component of the small ubiquitin-associated modifiers (SUMOs) E3 ligase, was activated by SIRT1 and it was indispensable for FTO SUMOylation at Lysine (K)-216 site that promotes FTO degradation [39]. Our in vitro results indicated that irisin-mediated SIRT1 could decrease FTO protein expression and inhibit adipogenic differentiation of BMMSCs and that SIRT1 did not specifically bind to FTO proteins. However, irisin-activated SIRT1 enhances the expression of RANBP2 through deacetylation function, and RANBP2 summoylation modifies FTO and reduces the stability of FTO.

The acetylation-modifying protein SIRT1, SUMO-modifying protein RANBP2, and Fat mass and obesity-associated protein FTO are progressively presented in irsin-mediated adipogenic differentiation of BMMSCs. Nevertheless, we have only begun to provide fundamental insights into irsin-mediated adipogenic differentiation of BMMSCs, a signaling pathway that facilitated the progression of OP resolution. Based on these findings, a very promising area of research would be potential diagnostic and therapeutic biomarkers for irsin, SIRT1, RANBP2, and FTO. In addition, a better understanding of the complex network of transcriptomics and epigenetic regulation in which irsin-mediated SIRT1 is involved could reveal new therapeutic strategies for OP patients.

Several limitations of this study warrant consideration. First, while we employed OVX mice model for postmenopausal osteoporosis, it is important to note that inherent species differences and the complexity of human disease pathophysiology may limit direct translation of these findings to clinical settings. Second, although our analysis of bone tissue samples from 20 patients (10 with osteoporosis and 10 without osteoporosis) provided preliminary evidence supporting the association between irisin and osteoporosis severity, the relatively small clinical sample size necessitates cautious interpretation of these results. In the future, we will conduct further research by increasing the number of clinical samples and through preclinical animal models and human clinical trials.

## Conclusions

**Fig. 7:**
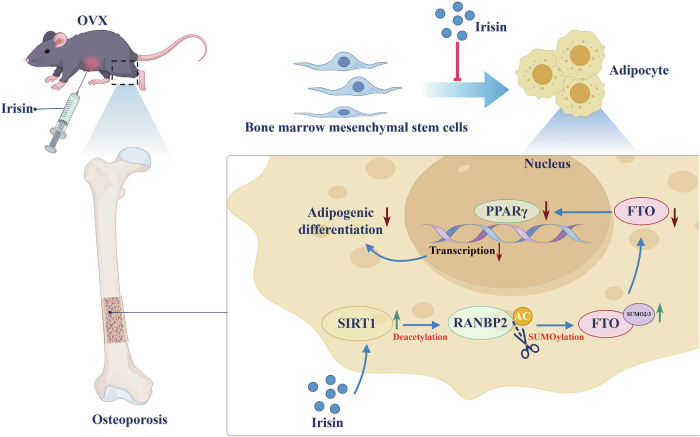
Irisin-mediated SIRT1 destabilizes FTO through the deacetylation of RANBP2, thereby inhibiting the adipogenic differentiation of bone marrow mesenchymal stem cells (BMMSCs), suggesting that the irisin/SIRT1/RANBP2/FTO signaling axis may serve as a new target for therapeutic drugs in OP.

We demonstrated an important mechanism by which irisin-mediated SIRT1 inhibited adipogenic differentiation of BMMSCs by destabilizing FTO through deacetylation of RANBP2 (Fig. 7). Our findings revealed the irisin/SIRT1/RANBP2/FTO signaling axis as a novel target for therapeutic OP drugs.

## Materials and methods

### Primary BMMSCs isolation

Primary BMMSCs were isolated from the femoral bone marrow of C57BL/6J mice following well-validated standard protocols. Briefly, murine femora were harvested, and both epiphyseal ends were carefully excised to expose the bone marrow cavity. Bone marrow contents were then gently flushed into 10-cm cell culture dishes using a syringe fitted with a 25G needle. The flushing and subsequent culture were conducted in α-minimum essential medium (12571063, Gibco, Grand Island, NY, USA) supplemented with 10% fetal bovine serum (FBS, F101, Vazyme, Nanjing, Jiangsu, China) and 1% penicillin-streptomycin (PS, 15140122, Gibco, Grand Island, NY, USA). All cultures were maintained in a humidified incubator at 37 °C with 5% CO₂. To eliminate non-adherent cells, half-volume medium replacement was performed daily. When the adherent cells reached ~90% confluency, they were subjected to trypsinization for passage.

### RNA sequencing transcriptomics and bioinformatics analyses

Total RNA was isolated from normal BMMSCs, adipogenically induced BMMSCs, and irisin-treated BMMSCs following adipogenic induction using TRIzol reagent (Invitrogen #15596026, USA), with 3 biological replicates per group. RNA quantity and purity were analyzed with Bioanalyzer 2100 and RNA 6000 Nano LabChip Kit (Agilent, CA, USA, 5067-1511), and high-quality RNA samples with RIN number >7.0 were used to construct the sequencing library. The library was sequenced on the Illumina Novaseq 6000 system (LC-Bio Technologies, China). Differential gene expression analysis was conducted in DESeq2 using the Wald test, applying thresholds of |FC| > 1 and FDR-adjusted *p* < 0.05. Data visualization using OmicStudio tools (https://www.omicstudio.cn/tool).

### Patient recruitment and data collection

The study was approved by the Medical Ethics Committee of Qilu Hospital (Qingdao), Shandong University (No. KYLL-2024035). Specimens were obtained from patients with diagnosed osteoporosis and controls without osteoporosis or other bone-related abnormalities. All subjects with other medical conditions and a history of smoking or alcohol consumption were excluded. All participants signed a written informed consent. Specimen collection was performed by the Department of Orthopedics, Qilu Hospital (Qingdao), Shandong University.

BMD for the lumbar spine and bilateral hips was measured by DXA. The *T*-value was commonly used to diagnose osteoporosis, which was calculated by an average value of Chinese young women. According to the WHO criteria, osteoporosis is diagnosed when BMD is 2.5 standard deviations below the average value of young individuals (*T*-score < −2.5). Blood was taken via venipuncture at 7:00 am in the fasting state (≥10 h). The serum was isolated and preserved at a temperature of −80 °C until use after centrifugation.

### Enzyme-linked immunosorbent assay (ELISA)

In this study, the serum concentrations of type I collagen cross-linked carboxyterminal telopeptide (CTX1) and irisin were measured using an ELISA kit (#CSB-EQ027943HU, cusabio, Wnhan, China) according to the manufacturer’s instructions.

### Animals

The experimental protocols of the mice were followed by the National Institute of Health’s Guide for the Care and Use of Laboratory Animals and permitted by the Laboratory Animal Ethical and Welfare Committee of Shandong University Cheeloo College of Medicine (No. 24014). All 12-week-old female C57BL/6 mice (6 mice per group) were purchased from the Beijing Vital River Laboratory Animal Technology Co., Ltd. After 1 week of adaptive feeding, mice were randomly divided into 4 groups (Sham group, OVX group, OVX + PBS group and OVX+Irisin group). OVX mice were operated bilaterally ovariectomized, which were surgically extracted by making a midline incision on the skin and flank incisions on the peritoneum. Mice in the sham group underwent a similar procedure, with only the fat tissue around the ovaries removed. The mice of the OVX+Irisin group were treated with an intraperitoneal injection of irisin with a dose of 100 μg/kg once a week, while the control group was intraperitoneally injected with the same dose of PBS. For the Irisin+selisistat(#HY-15452, MedChemExpress, USA) group, the mice were intraperitoneally injected with selisistat at a dose of 10 mg/kg once weekly.

### Micro-computed tomography (micro-CT) and analysis

The micro-CT system (PerkinElmer, USA) was used to scan the distal femur. Bones were scanned at a high resolution (7 μm/pixel) with an energy of 90 kV and 88 μA of intensity. Three-dimensional (3D) reconstruction of CT scan data and analysis of bone-related parameters: Cortical bone volume/tissue volume (Ct. BV/TV), trabecular bone volume/tissue volume (Tb. BV/TV), trabecular thickness (Tb.Th), trabecular number (Tb.N), trabecular separation (Tb.Sp), cortical bone thickness (Ct.Th), were performed according to previous research methods [42].

### Histological and immunological staining

Femoral samples were decalcified in 10% ethylenediaminetetraacetic acid (EDTA, pH 7.6) for 2 weeks after micro-CT analysis. The samples were immersed in 20% sucrose solution overnight at 4 °C and then embedded in (optimal cutting temperature) OCT complex to prepare frozen sections (10 μm). For paraffin sections (5 μm), samples were dehydrated using degraded ethanol and xylene according to previous work [42].

Hematoxylin-eosin (H&E) staining, Masson staining, and immunohistochemistry staining were performed according to previous work [58].

Frozen sections were stained with Oil Red O solution to evaluate adipose tissue in bone marrow. Frozen sections were pretreated with 60% isopropyl alcohol for 10 min, stained with 3% Oil Red O solution for 15 min, and stained with hematoxylin for 1 min. Images were taken under the microscope (Olympus, Japan).

### Cell counting kit-8 (CCK-8)

BMMSCs were seeded in 96-well plates at 2 × 10^3^ cells/well and treated with various concentrations of recombinant irisin (0, 25, 50, 75, 100, and 125 ng/ml) (#HY-P70665, MCE, China), respectively. Cell proliferation was assessed on hours 12, 24, 36, and 48 using the CCK-8 assay (#E-CK-A362, Elabscience, China). Optical density (OD) was measured at 450 nm (Bio-Rad, USA).

### Alkaline phosphatase (ALP) and alizarin red S (ARS) staining

After osteogenic induction, BMMSCs were fixed with 4% paraformaldehyde for 20 min. After rinsing three times with Phosphate Buffer Saline (PBS), ARS staining solution (#G1450, Solarbio, China) was stained for 20 min and finally washed three times with ddH2O. ALP staining was performed using BCIP/NBT Alkaline Phosphatase Chromogenic Kit (#C3206, Beyotime, China). The results of ARS staining and ALP staining were captured as images by microscope (Zeiss, Germany). Quantification was performed using ImageJ software (NIH, Bethesda, MD, USA).

### Quantitative real-time polymerase chain reaction

The TRIzol reagent (#15596026, Invitrogen, USA) was used to isolate the RNA of all the cells. Reverse transcription and Quantitative real-time polymerase chain reaction experimental manipulations (qRT-PCR) were performed as described previously [42]. GAPDH was used as an internal reference and normalized by the comparative CT method. Primers used refer to Table S1.

### Western blotting

Western blotting was performed based on previous studies [58]. In brief, equal amounts of protein samples were separated on polyacrylamide gels and transferred to nitrocellulose membranes, which were closed for 1 h using 5% skimmed milk powder, and the membranes obtained were incubated with anti-FNDC5 antibody (#23995-1-AP, 1:1000, Proteintech, China), anti-PPARγ antibody (#7273, 1:1000, Santa Cruz Biotechnology, USA), anti-C/EBPα antibody (#2295, 1:1000, Cell Signaling Technology, USA), anti-C/EBPβ antibody (#3087, 1:1000, Cell Signaling Technology, USA), anti-SIRT1 antibody (#8469, 1:1000, Cell Signaling Technology, USA), anti-FTO antibody (#27226-1-AP, Proteintech, China), anti-RANBP2 antibody (#ab315458, 1:1000, Abcam, USA), and anti-GAPDH antibody (#10494-1, 1:1000, proteintech, China) were incubated at 4 °C overnight. The treated membranes were incubated with a secondary antibody. Finally, the antibody-antigen complexes were observed with a Tanon 5200 chemiluminescence image analysis system (Tanon, China).

For co-immunoprecipitation, total cellular proteins were extracted using lysis buffer, and then, the extracts were incubated with anti-FTO antibody (#27226-1-AP, Proteintech, China), anti-SIRT1 antibody (#8469, 1:1000, Cell Signaling Technology, USA) or IgG at 4 °C overnight. Then the mixture was incubated with Protein A&G beads (#B23201, Selleck, Germany) at 4 °C overnight. The coprecipitated proteins were washed four times with lysis buffer and analyzed by Western blotting with designated antibodies.

### Oil red O staining

Two weeks after induction of adipogenesis, bmmsc-derived adipocytes were fixed with 4% formaldehyde and then incubated with Oil Red O stain. Adipocytes were recorded with a microscope (Zeiss, Germany).

### siRNA and plasmid transfection

Mouse *Sirt1*-siRNA, *Ranbp2*-siRNA, and negative control (si-NC) were obtained from GenePharma Co., Ltd (Shanghai, China). siRNA was diluted with DEPC water. For single wells of 6-well plates, 100 pmol siRNA and 5 μL of Liposome 3000 Transfection Reagent (#L3000075, Invitrogen, USA) were added to 250 μL of Opti-MEM I (#11058021, Invitrogen, USA) and thoroughly mixed, and left for 5 min at room temperature. For transfection, the medium was replaced with 1.5 mL of Opti-MEM I. After 4 h, the medium was replaced with fresh medium. Subsequent experiments were performed 48 h after transfection. Primers used refer to Table S2.

The process of OE *Sirt1* plasmid and OE *Fto* plasmid construction was referred to in the previous study [52]. The amount of plasmid was 4 μg, and the amount of liposome 3000 transfection reagent was 10 μL.

### Immunofluorescent (IF) staining

Pretreated cells were rinsed 3 times with PBS, fixed with 4% paraformaldehyde and permeabilized with 0.1% Triton X-100 (Beyotime, China) for 10 min. The cells were then blocked with 5% Bovine Serum Albumin (BSA) for 30 min, incubated in primary antibody against SIRT1(#8469, 1:1000, Cell Signaling Technology, USA) and RANBP2 (#ab315458, 1:1000, Abcam, USA) overnight at 4 °C, and secondary antibody for 45 min. Nuclei were stained with DAPI. Images were taken under confocal microscopy (Olympus, Japan).

### Statistical analysis

Quantitative data are expressed as mean ± standard deviation (SD), where *n* denotes: the number of animals per group in in vivo experiments, or the number of independent replicates in in vitro experiments. Sample sizes were determined based on preliminary experimental data. For in vitro experiments, each experiment was independently repeated at least 3 times. For animal experiments, a minimum of 6 biological replicates were set per group. The Shapiro–Wilk test was used to verify the data distribution type. Comparisons between two groups were performed using an unpaired two-tailed Student’s *t*-test, differences among multiple groups were analyzed by one-way analysis of variance followed by appropriate post hoc tests. Scatter plots with bars were used to describe the overall data distribution without assuming the statistical distribution type of the data. All statistical analyses were performed using GraphPad Prism software (Version 9.0.0, Boston, MA, USA). Investigators remained blinded to group assignments, and all experimental subjects were randomly allocated throughout the study. No experimental subjects, biological samples, or data points were excluded in this study.

## Supplementary information

Table S1

Table S2

Table S3

Figure S1

Figure S2

Figure S3

Figure S4

Figure S5

Original western blots

## Data Availability

All relevant data and materials are available from the authors upon reasonable request.
