# Supplementary material for: Irisin inhibits adipogenic differentiation of bone marrow mesenchymal stem cells through the SIRT1/RANBP2/FTO signaling axis and protects against osteoporosis
Source: Cell Death Discov. 2026 Feb 25;12:114. doi: 10.1038/s41420-026-02976-5 (PMC12988873; doi:10.1038/s41420-026-02976-5)
Supplement: Supplementary file 1 — Table S1 [file 41420_2026_2976_MOESM1_ESM.docx]

**Table S1. Primer sequences used for quantitative real-time PCR**

| **Gene** | **Forward Primers,5’-3’** | **Reverse Primers,5’-3’** |
| --- | --- | --- |
| ***Gapdh*** | TGTCTCCTGCGACTTCAACA | GGTCGTCCAGGGTTTCTTACT |
| ***Bmp2*** | TGTCCCCAGTGACGAGTTTC | TCGAAGCTCTCCCACTGACT |
| ***Col1*** | CCCTGGTCCCTCTGGAAATG | GGACCTTTGCCCCCTTCTTT |
| ***Pparγ*** | GCCGAGTCTGTGGGGATAAA | TCCGGCAGTTAAGATCACACC |
| ***C/ebpα*** | AGGAACACGAAGCACGATCAG | CGCACATTCACATTGCACAA |
| ***C/ebpβ*** | CTTCAGCCCGTACCTGGAG | GGAGAGGAAGTCGTGGTGC |
| ***Serpina3n*** | TGTCTGCGAAACTGTACCCTC | GGGGTTGGCTATCTTGGCT |
| ***Nos2*** | CCTGCTTTGTGCGAAGTGTC | CCCAAACACCAAGCTCATGC |
| ***Rdh9*** | GCCTTTGGACTCTCCTGTGCTT | AGACATGCAGCCAACACTCTC |
| ***Serpina3m*** | TGAGTGTGTCTCAGGTGGTC | GAAAGCCAAAAATGAACCCTGT |
| ***Sirt1*** | TATCTATGCTCGCCTTGCGG | GACACAGAGACGGCTGGAAC |
| ***Fto*** | GACGTGGTGAGGATCCAAGG | AGCCTCTGTGTACTTGACCG |
| ***Alkbh5*** | CCGTGTCTTTCTTCAGCGAC | TAATGTCCTGAGGCCGTATGC |
| ***Ranbp2*** | TTGCCTGTTCCCCTTGAGTC | CACTTCGCCAGCAACCATTT |
