# Supplementary material for: Irisin inhibits adipogenic differentiation of bone marrow mesenchymal stem cells through the SIRT1/RANBP2/FTO signaling axis and protects against osteoporosis
Source: Cell Death Discov. 2026 Feb 25;12:114. doi: 10.1038/s41420-026-02976-5 (PMC12988873; doi:10.1038/s41420-026-02976-5)
Supplement: Supplementary file 2 — Table S2 [file 41420_2026_2976_MOESM2_ESM.docx]

**Table S2. Primers used for si-RNA**

|  | | |
| --- | --- | --- |
| **Gene** | **Forward Primers,5’-3’** | **Reverse Primers,5’-3’** |
| ***si-NC*** | UUCUCCGAACGUGUCACGUTT | ACGUGACACGUUCGGAGAATT |
| ***si-Sirt1-1*** | GGUUGUUAAUGAAGCUAUATT | UAUAGCUUCAUUAACAACCTT |
| ***si-Sirt1-2*** | GAGUCCAAGUUUAGAAGAATT | UUCUUCUAAACUUGGACUCTT |
| ***si-Sirt1-3*** | CCCAUGAAGUGCCUCAAAUTT | AUUUGAGGCACUUCAUGGGTT |
| ***si-Ranbp2-1*** | GGAGGAUGAUGAAGAUUAUTT | AUAAUCUUCAUCAUCCUCCTT |
| ***si-Ranbp2-2*** | GGGCACAUGUUGUUAAACUTT | AGUUUAACAACAUGUGCCCTT |
| ***si-Ranbp2-3*** | GGGCCUUACUGGAAUUGUATT | UACAAUUCCAGUAAGGCCCTT |
