# Supplementary material for: Irisin inhibits adipogenic differentiation of bone marrow mesenchymal stem cells through the SIRT1/RANBP2/FTO signaling axis and protects against osteoporosis
Source: Cell Death Discov. 2026 Feb 25;12:114. doi: 10.1038/s41420-026-02976-5 (PMC12988873; doi:10.1038/s41420-026-02976-5)
Supplement: Supplementary file 3 — Table S3 [file 41420_2026_2976_MOESM3_ESM.docx]

**Table S3. Baseline characteristics of study participants.**

|  | Non-OP (n=10) | OP (n=10) | P value |
| --- | --- | --- | --- |
| Age | 59.8±10.2 | 59.7±10.0 | 0.983 |
| Height (cm) | 163±5.95 | 163±3.89 | 0.896 |
| Weight (kg) | 59.2±9.61 | 61.9±8.03 | 0.504 |
| BMI (kg/m^2^) | 21.2±1.92 | 21.5±3.20 | 0.767 |
| L1-L4 Bone Mineral Density T-Score | -0.61±0.22 | -3.52±0.80 | <0.001 |
| Hip Bone Mineral Density T Score | -0.87±0.23 | -3.62±0.63 | <0.001 |

Quantitative variables are reported as means ± standard deviations (SD). p-values for quantitative variables were calculated using the Student’s t-test to assess significance.
