# Supplementary material for: Irisin inhibits adipogenic differentiation of bone marrow mesenchymal stem cells through the SIRT1/RANBP2/FTO signaling axis and protects against osteoporosis
Source: Cell Death Discov. 2026 Feb 25;12:114. doi: 10.1038/s41420-026-02976-5 (PMC12988873; doi:10.1038/s41420-026-02976-5)

**Figure S1.** (A) *Sirt1* mRNA expression in *Sirt1*-overexpressed cells was assayed by qRT-PCR. SIRT1 protein expression in *Sirt1*-overexpressed cells was assayed by western blotting. (B) mRNA expressions of *Pparγ*, *C/ebpα*, and *C/ebpβ* in *Sirt1* knockdown or *Sirt1*-overexpressed cells was assayed by qRT-PCR. (C) Immunoblotting of adipogenic related proteins (PPARγ, C/EBPα, and C/EBPβ) in *Sirt1* knockdown or *Sirt1*-overexpressed cells was assayed by western blotting. (D) Representative images of Oil red O staining in *Sirt1* knockdown or *Sirt1*-overexpressed cells, and quantitative analysis of Oil red O staining. The values are mean ± SD of at least three independent experiments; ^n.s.^p>.05, ^*^p<.05, ^**^p<.01, ^***^p<.001, ^****^p < .0001.


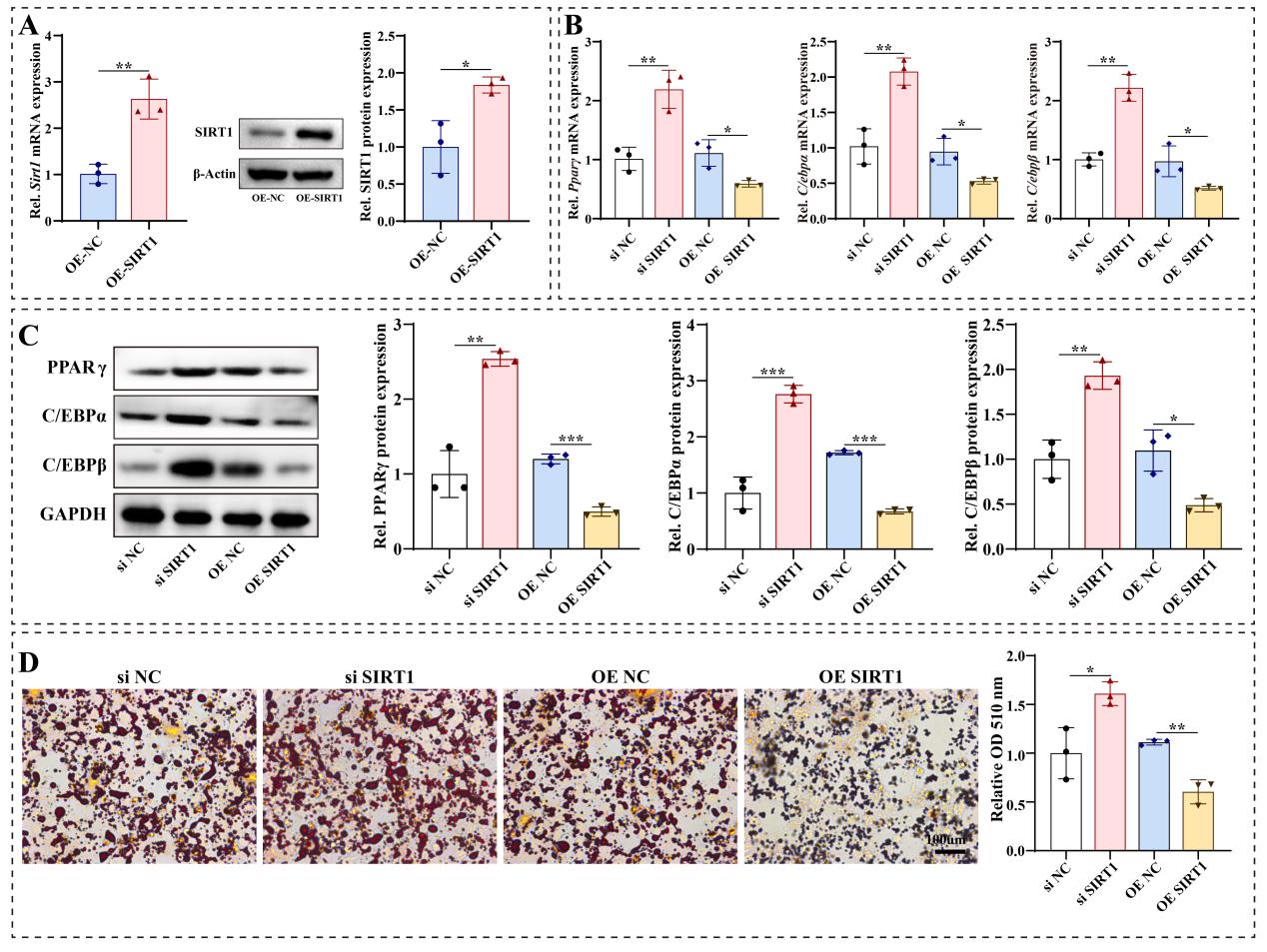

Supplement: Supplementary file 4 — Figure S1 [file 41420_2026_2976_MOESM4_ESM.docx]
