# Supplementary material for: Irisin inhibits adipogenic differentiation of bone marrow mesenchymal stem cells through the SIRT1/RANBP2/FTO signaling axis and protects against osteoporosis
Source: Cell Death Discov. 2026 Feb 25;12:114. doi: 10.1038/s41420-026-02976-5 (PMC12988873; doi:10.1038/s41420-026-02976-5)

**Figure S2.** (A) qRT-PCR was performed to detect the expression level of *Sirt1* mRNA in cells treated with PBS, irisin, and cilengitide. ^n.s.^p>.05, ^*^p<.05, ^**^p<.01, ^***^p<.001, ^****^p < .0001.


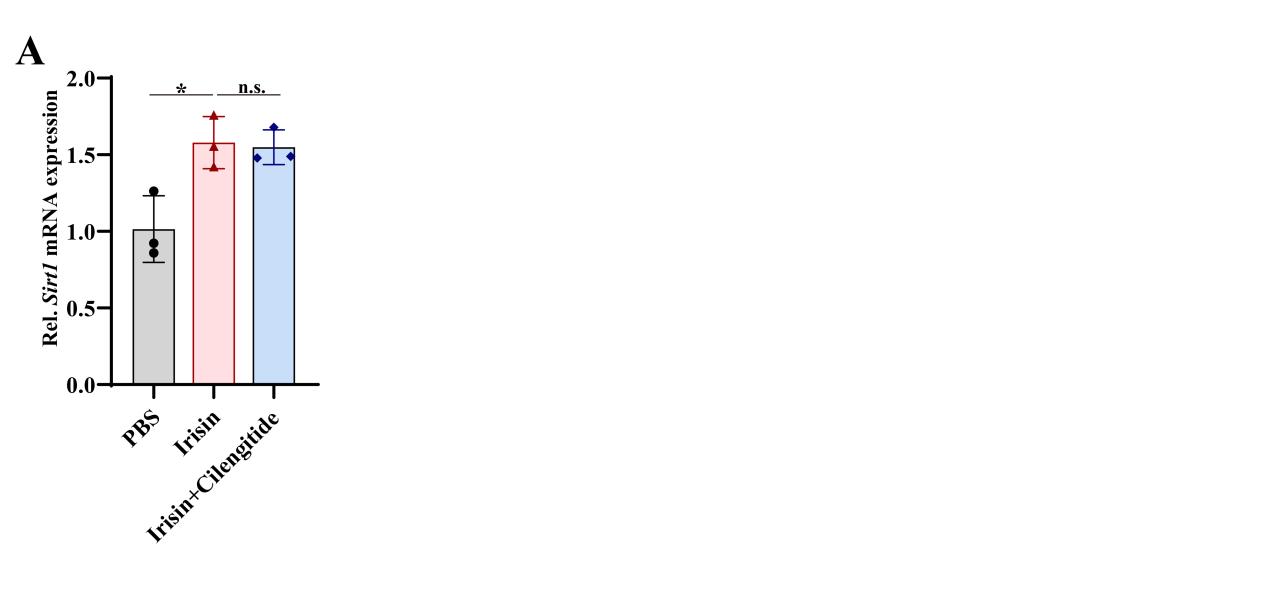

Supplement: Supplementary file 5 — Figure S2 [file 41420_2026_2976_MOESM5_ESM.docx]
