# Supplementary material for: Irisin inhibits adipogenic differentiation of bone marrow mesenchymal stem cells through the SIRT1/RANBP2/FTO signaling axis and protects against osteoporosis
Source: Cell Death Discov. 2026 Feb 25;12:114. doi: 10.1038/s41420-026-02976-5 (PMC12988873; doi:10.1038/s41420-026-02976-5)

**Figure S3.** (A) Representative micro-CT images of trabecular bone from the femoral metaphysis in mice treated with irisin and selisistat. Cortical bone volume/total volume (Ct. BV/TV), trabecular bone volume/total volume (Tb. BV/TV), trabecular thickness (Tb. Th), cortical thickness (Ct. Th), and number of trabeculae (Tb. N), and trabecular separation (Tb. Sp) analysis of the femurs in mice. (B) Representative images of Oil red O staining in the distal femur, and quantitative analysis of Oil red O staining. ^n.s.^p>.05, ^*^p<.05, ^**^p<.01, ^***^p<.001, ^****^p < .0001.


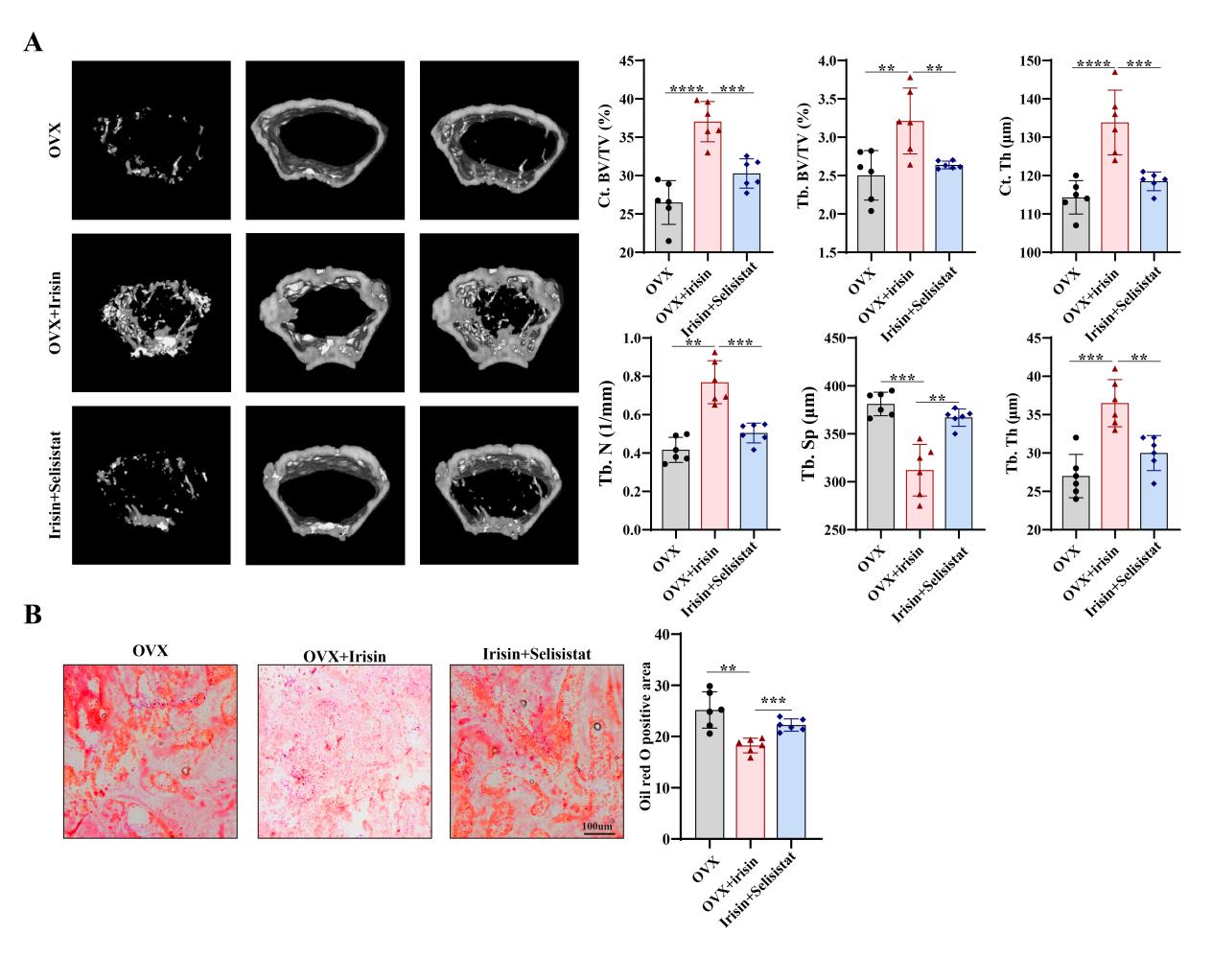

Supplement: Supplementary file 6 — Figure S3 [file 41420_2026_2976_MOESM6_ESM.docx]
