# Supplementary material for: Irisin inhibits adipogenic differentiation of bone marrow mesenchymal stem cells through the SIRT1/RANBP2/FTO signaling axis and protects against osteoporosis
Source: Cell Death Discov. 2026 Feb 25;12:114. doi: 10.1038/s41420-026-02976-5 (PMC12988873; doi:10.1038/s41420-026-02976-5)

**Figure S4.** (A) *Fto* mRNA expression in *Fto*-overexpressed cells was assayed by qRT-PCR. FTO protein expression in *Fto*-overexpressed cells was assayed by western blotting. (B) mRNA expressions of *Pparγ*, *C/ebpα*, and *C/ebpβ* in *Sirt1*-overexpressed cells with or without *Fto* overexpression was assayed by qRT-PCR. The values are mean ± SD of at least three independent experiments; ^n.s.^p>.05, ^*^p<.05, ^**^p<.01, ^***^p<.001, ^****^p < .0001.


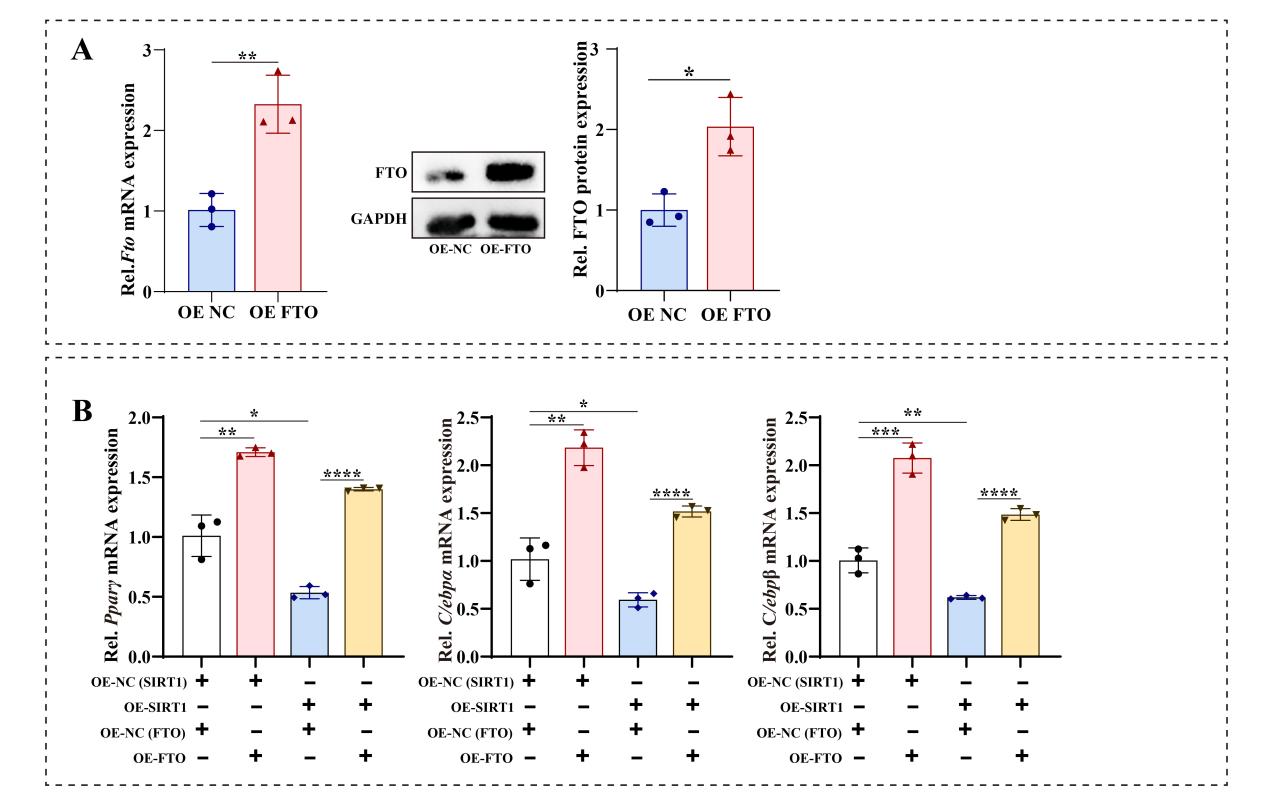

Supplement: Supplementary file 7 — Figure S4 [file 41420_2026_2976_MOESM7_ESM.docx]
