# Supplementary material for: Irisin inhibits adipogenic differentiation of bone marrow mesenchymal stem cells through the SIRT1/RANBP2/FTO signaling axis and protects against osteoporosis
Source: Cell Death Discov. 2026 Feb 25;12:114. doi: 10.1038/s41420-026-02976-5 (PMC12988873; doi:10.1038/s41420-026-02976-5)

**Figure S5.** (A) *Ranbp2* mRNA expression in *Sirt1* knockdown cells with or without irisin treatment was assayed by qRT-PCR. ^n.s.^p>.05, ^*^p<.05, ^**^p<.01, ^***^p<.001, ^****^p < .0001.


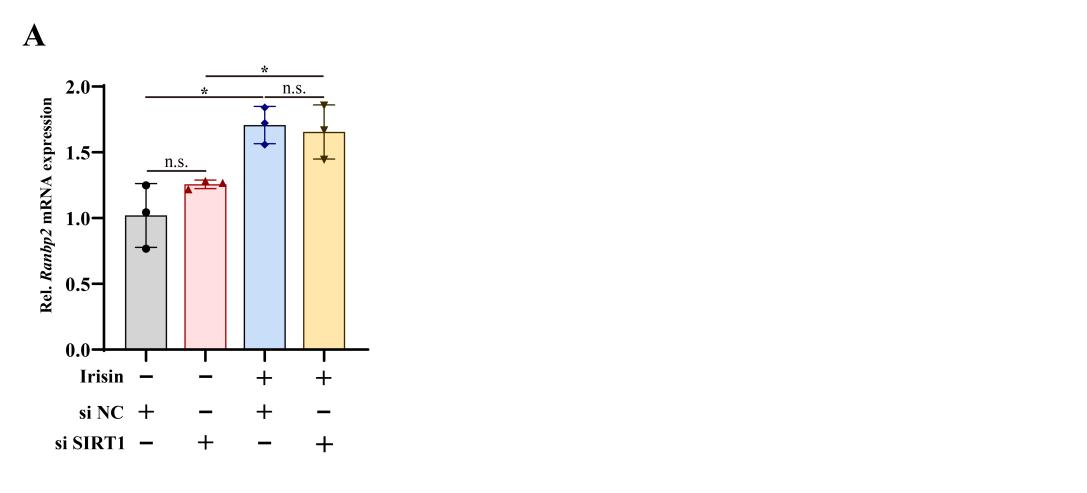

Supplement: Supplementary file 8 — Figure S5 [file 41420_2026_2976_MOESM8_ESM.docx]
