# Supplementary material for: Irisin inhibits adipogenic differentiation of bone marrow mesenchymal stem cells through the SIRT1/RANBP2/FTO signaling axis and protects against osteoporosis
Source: Cell Death Discov. 2026 Feb 25;12:114. doi: 10.1038/s41420-026-02976-5 (PMC12988873; doi:10.1038/s41420-026-02976-5)

Figure 1J

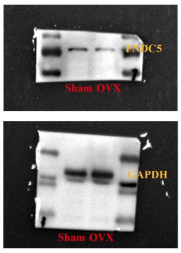

Figure 2I

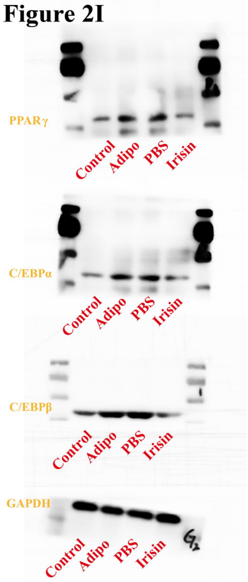

Figure 4E

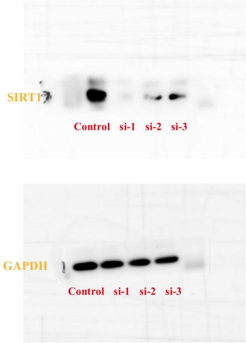

Figure 4G

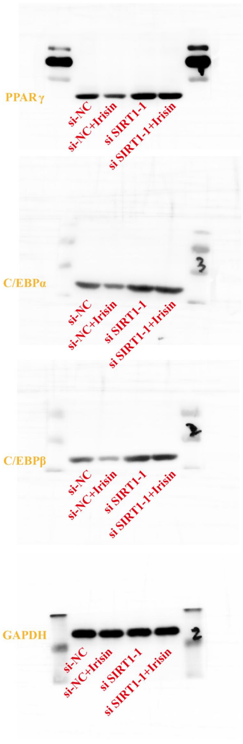

Figure 5B

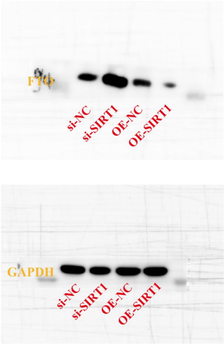

Figure 5C

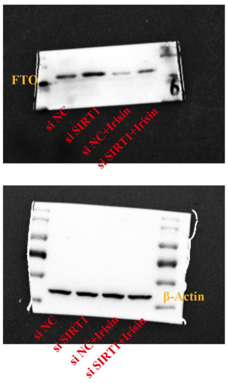

Figure 5D

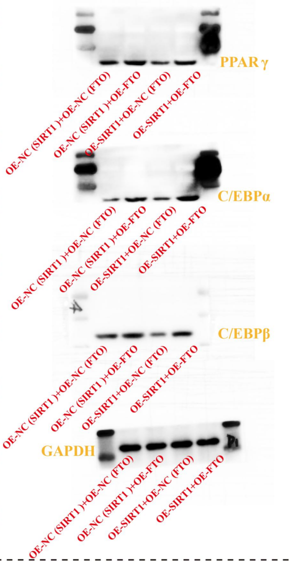

Figure 6A

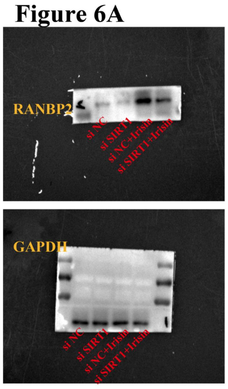

Figure 6B

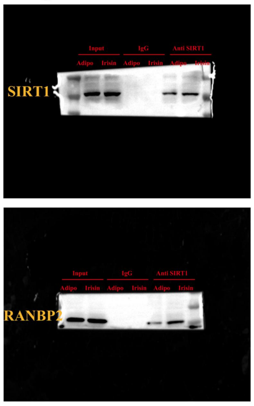

Figure 6G

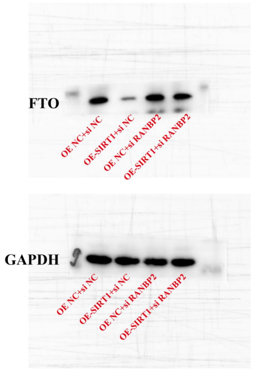

Figure 6H

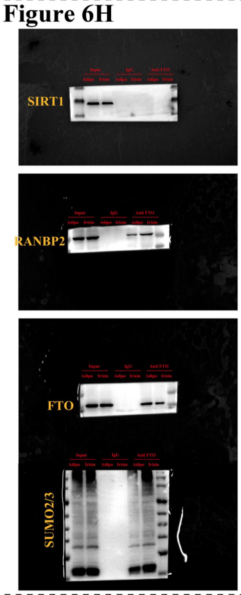

Figure S1A

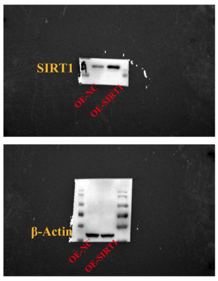

Figure S1C

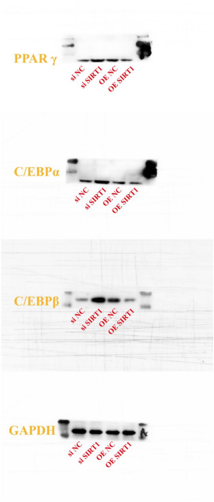

Figure S2A

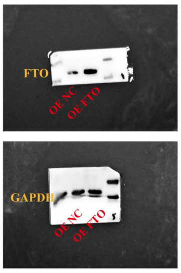

Supplement: Supplementary file 9 — Original western blots [file 41420_2026_2976_MOESM9_ESM.pdf]
